# Mineral element concentrations of common grass and shrub species on sheep winter range in Wyoming: insights for mineral supplementation strategies^1^

**DOI:** 10.1093/tas/txaa088

**Published:** 2020-12-22

**Authors:** Alexis A M Julian, John D Scasta, Barton R Stam, Brian M Sebade, Chad M Page, Brady E Springer, Wilson T Renner, Hannah Cunningham-Hollinger, Whitney C Stewart

**Affiliations:** 1 Department of Animal Science, University of Wyoming, Laramie, WY; 2 Department of Ecosystem Science and Management, University of Wyoming, Laramie, WY; 3 University of Wyoming Extension, Thermopolis, WY; 4 University of Wyoming Extension, Laramie, WY

## INTRODUCTION

Rangelands are natural ecosystems that include an inherent diversity of native grasses, forbs, and shrubs all of which are commonly utilized by livestock and wildlife. In Wyoming, United States, approximately 85% of the land surface area is considered rangelands. Plant communities on these rangelands span from sagebrush steppe to shortgrass prairie and include plant communities important for sheep production such as salt desert shrublands (Knight et al., 2014). As of 2017, Wyoming’s total sheep inventory is approximately 367,702 (USDA-NASS, 2017), to which 82% come from ranches with 1,000 sheep or more. Furthermore, we estimate that approximately 82% of all Wyoming sheep come from operations that utilize winter range resources.

Sheep rely heavily on rangelands and depend on dormant forages to provide their macro- and micronutrient requirements. Forage mineral elements naturally vary in concentrations due to a multitude of factors including soil fertility, plant phenology, and land management (Spears, 1994; Smith et al., 2014; Jones and Tracy, 2015). Additionally, mineral element concentrations in a single region and/or in a major feed category have been found to be extremely variable (Adams, 1975; Mathis and Sawyer, 2004; NRC, 2007) and can result in ewes with clinical and subclinical deficiencies in extensive production settings.

Likely unnoticed, trace mineral deficiencies can cause significant economic impacts to producers. Therefore, supplementation strategies and nutritional management of ewes during critical production periods of breeding and gestation are an important consideration when managing ewes on winter range. Page et al. (2018) found that 33% of Montana sheep producers were not supplementing a complete trace mineral, and of those trace minerals Se and Zn represented mineral elements most commonly deficient and marginally deficient, thus there is room for improvement in this area.

While existing scientific literature has quantified sheep dietary composition of forages on winter range in Wyoming and the nutritional composition of common winter range forages (Severson and May, 1967; Ngugi et al., 1992), there is a lack of information for trace mineral composition of dormant forages. Thus, the objectives of this study were to 1) quantify mineral element concentrations of common forages on winter range and 2) evaluate producer supplementation strategies. We hypothesized that mineral element concentrations in forages were inadequate to meet ewe requirements during critical production stages and shrub species would contain higher levels of macro- and micromineral concentrations when compared with grass species.

## MATERIALS AND METHODS

**Figure 1.**
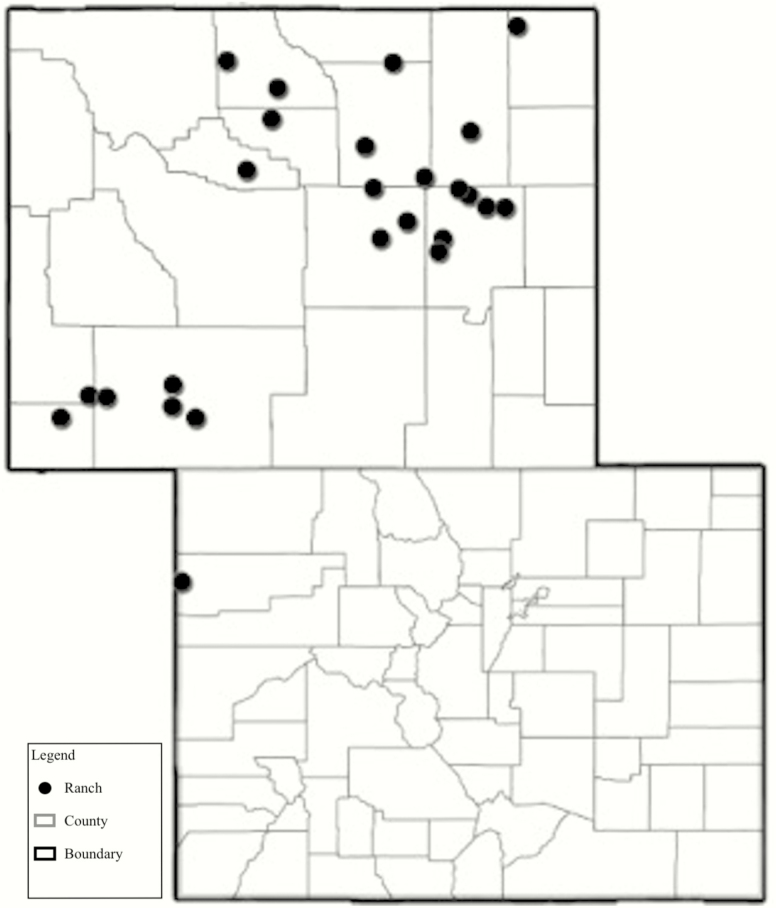
Map of winter range sampling locations across Wyoming and Colorado.

From December through February of 2018 and 2019, forage samples were collected on 25 winter ranges across the state of Wyoming (Fig. 1) in order to quantify trace minerals of common forages in sheep winter range and examine mineral variability across sampling sites. Data presented only include mineral element data from 12 of 25 ranches (year 1 of 2) due to concurrent analyses. Sheep producers participating in the study were invited to complete a survey with 19 ranches of the 25 ranches responding. The survey which covered production practices, and related nutritional management, with a total of 36 questions of which only 6 questions are contained herein. At each ranch, winter range sites sampling locations (plots; *n* = 3) were determined relative to where sheep were grazing winter range. Plots were spatially separated and representative of the individual producer’s winter range. Global Positioning System coordinates and elevation were recorded at each plot. A minimum of eight plants for each species were sampled within each plot. Individual plant species across three plots were then composited and stored at −20 °C until laboratory analysis. The plant sampling protocol involved plucking plant material to simulate the selectivity of sheep grazing behavior as found by Cook et al. (1948). Forage species across ranches included blue grama (*Bouteloua gracilis*), needle-and-thread (*Hesperostipa comata*), prairie junegrass (*Koeleria macrantha*), prairie sandreed (*Calamovilfa longifolia*), sand dropseed (*Sporobolus cryptandrus*), western wheatgrass (*Pascopyrum smithii*), and Indian ricegrass (*Oryzopsis hymenoides*). Shrub species sampled included Wyoming big sagebrush (*Artemisia tridentata* ssp. *wyomingensis*), rubber rabbitbrush (*Ericameria nauseosa*), Gardner’s saltbush (*Atriplex gardneri*), shadscale saltbush (*Atriplex confertifolia*), silver sagebrush (*Artemisia cana*), and winterfat (*Krascheninnikovia lanata*).

### Laboratory Analysis

Dry matter of ground grass and shrub species was calculated by drying ground material at 64 °C in a forced air oven for 24 h. Material was weighed and then dried again at 105 °C for 3 h. Nitrogen was analyzed (Method 990.03; AOAC, 2006; Leco Corp., St. Joseph, MI) and crude protein (CP) was derived relative to N concentration. For mineral analyses, Organic Matter and lipids were removed from samples via HNO_3_, HCl, and H_2_O_2_ (Campbell et al., 1991) and then analyzed for each mineral by inductively coupled plasma spectroscopy (Kovar, 2003).

### Statistical Analysis

Data were analyzed in R using the general linear model procedure and are presented as least square means (R Core Team, 2020). Descriptive statistics (mean ± SD) of nutrient and mineral element concentrations of each grass and shrub species were estimated among all ranches combined using the Least Squares Mean procedure in R. To determine the effect of forage type (e.g., grass vs. shrub) on nutrient and mineral element concentrations, forage species within ranch was the experimental unit (shrub *n* = 40; grass *n* = 30), and the mean transformed forage mineral concentrations were analyzed in the General Linear Models procedure with the fixed effect of forage type (shrub or grass). Differences among means were considered significant at the 95% confidence level (*α* < 0.05).

## RESULTS AND DISCUSSION

A total of 19 sheep producers participated in the survey questionnaire and responses are summarized in Table 1. Total number of ewes managed on winter range consisted of flock sizes ranging from 450 to 12,000 head. Of the surveys completed, approximately 84% (16 responses) of sheep come from ranches with ≥1,000 sheep utilizing winter range. Three respondents or 16% of sheep come from ranches ≤999 sheep but are also utilizing winter range. Ewes managed on winter range represented a significant part of the production year for operations surveyed (e.g., 26%, 90 to 130 d; 47%, 131 to 170 d; 26%, >171 d). Lower nutritional content of available forages on winter range coincided with critical production periods of breeding and gestation, which emphasizes the importance of informed supplementation strategies. According to the producer survey, 47% of respondents provide a complete trace mineral supplement while on winter range and 79% of producers had not tested their feeds in the past 5 yr (15 responses). Reasons for not providing a complete trace mineral varied but may be related to cost and return on investment and logistical concerns such as transient movement across winter ranges.

**Table 1.** Survey of production and nutritional practices of sheep producers in Wyoming (*n* = 19)

Item	Percentage
Total number of ewes managed on winter range	
≤1,000	21%
1,001–5,000	53%
5,001–9,000	16%
≥9,001	11%
Duration on winter range	
90–130 d	26%
131–170 d	47%
171+ d	26%
Energy^*a*^ vs. protein^*b*^ supplementation	
Energy	16%
Protein	47%
Energy and protein	21%
None	16%
White salt supplementation in winter	
Supplemented	37%
Unsupplemented	58%
Varies year to year	5%
Complete trace mineral mix or tub supplement	
Supplemented	47%
Unsupplemented	42%
Varies year to year	11%
Feeds tested in the past 5 yr	
Tested	21%
Untested	79%

^*a*^Energy supplements: corn and hay.

^*b*^Protein supplements: cake, alfalfa, and protein lick tubs.

To compare mineral concentrations in grass vs. shrub species, statistical comparisons are summarized in Table 2. When averaged across species, shrubs had significantly greater mineral element concentrations than grass species (all *P*-values < 0.03), with the exception of Mo and Mg which were lower in shrubs (2.08 vs. 1.34 and 0.09 vs. 0.05, respectively), and with the exception of Fe and Co which did not differ between grasses and shrubs (*P* = 0.37 and 0.29, respectively; Table 2). The magnitude of the differences for macrominerals, Ca, P, K, Mg, S, and Na, was all approximately 55% greater in shrubs (Table 2). Of these minerals, Na had the greatest relative difference at 97% in concentration from grass to shrub species (0.06% vs. 1.91%; *P* = 0.003). Microminerals, Zn, Cu, Se, and Mn, were all more than 40% greater in shrubs. It is important to note that mineral element concentrations in grass species were similar to those reported by Sprinkle et al. (2017) for grasses sampled in the fall.

**Table 2.** Macro- and micromineral concentrations in common forages collected winter range

	Grass^*a*^	Shrub^*b*^	SEM	*P*-value
Item, % DM				
CP	3.85	9.43	0.41	<0.001
Ca	0.47	1.48	0.10	<0.001
P	0.06	1.48	0.01	<0.001
K	0.19	1.20	0.10	<0.001
Mg	0.09	0.05	0.08	0.0001
S	0.09	0.30	0.03	<0.001
Na	0.06	1.91	0.36	0.0002
Item, mg/kg DM				
Zn	22.8	38.2	3.28	0.007
Cu	2.91	7.51	0.44	<0.001
Se	0.36	1.43	0.36	0.03
Mn	36.5	83.1	10.2	0.001
Mo	2.08	1.34	0.25	0.03
Fe	615	902	238	0.37
Co	0.30	0.46	0.11	0.29

DM = dry matter.

^*a*^Macro- and micromineral concentrations were averaged across all grasses (*n* = 30).

^*b*^Macro- and micromineral concentration were averaged across all shrubs (*n* = 40).

In shrub dominated rangeland environments, sheep diets consisting between 59% and 80% shrubs have been reported (Cook and Harris, 1950; Hutchings and Stewart, 1953). The diversity of forages observed on Wyoming rangelands varies from a shrub dominated plant communities to grass monocultures. Thus, assuming requirements for a 82 kg ewe carrying twins on a shrub dominated rangeland consuming 2% of her Body Weight in dormant forages would meet over 100% of K requirements for breeding, early gestation and late gestation (80% shrub vs. 20% grass intake). While assuming the same requirements, but for a ewe grazing a grass monoculture would meet 86%, 73%, and 69% of K requirements for breeding, early gestation, and late gestation, respectively (20% shrub vs. 80% grass intake). A similar effect was observed for S.

Crude protein concentrations of grass and shrub species are summarized in Table 3. Crude protein is important in optimizing the microbial synthesis. When CP is deficient, rumen microorganisms are impaired and digestion rate is slower (NRC, 2007). Moreover, CP, energy, phosphorous, and vitamin A are often the limiting nutrients in range livestock production systems (Holechek and Herbel, 1986). According to Table 1, 47% of producers were supplementing with a protein source (nine responses) and 16% supplementing with an energy source (three responses), while 16% were not providing an energy or protein supplement (three responses). Generally, CP was greater in shrubs (range of 6.03% to 12.66%) than grasses (range of 2.80% to 4.35%) (Table 3). The greatest CP of all shrubs was 12.66% in Gardner’s saltbush. The CP requirement for an 80 kg ewe carrying twins in late gestation is 10.9% (NRC, 2007), suggesting that ewes grazing sites with shrubs available could meet CP requirements. Similar results for CP concentrations between grasses and shrubs (Table 2) were found by Gade and Provenza (1986).

**Table 3.** Mean CP concentrations found in common forages collected from winter range^*a*^

	CP, %	±SD	# of ranches
Grass species			
Blue grama	3.13	0.67	3
Needle-and-thread	2.80	0.20	3
Prairie junegrass	3.17	0.15	3
Prairie sandreed	4.35	0.78	2
Sand dropseed	3.65	0.35	2
Western wheatgrass	4.00	1.18	8
Indian ricegrass	4.23	0.76	7
Shrub species			
Wyoming big sagebrush	11.03	0.86	11
Rabbitbrush	6.03	1.00	8
Gardner’s saltbush	12.66	2.41	8
Shadscale saltbush	8.45	1.95	6
Silver sagebrush	8.35	1.77	2
Winterfat	7.85	1.95	4

^*a*^Means and SD on a dry matter basis.

Macromineral concentrations of grass and shrub species are summarized in Table 4. Sodium and Cl when chemically combined are salt and the provision of salt in mineral supplements is used to manipulate intake (Underwood, 1981). Survey results show more than half of the producers (58%) do not provide white salt in the winter. Gardner’s saltbush has the highest concentration at 6.03% (Table 4). Daily salt requirements for gestating ewes approximates 2.0 g/d (NRC, 2007) and under grazing conditions with high saltbush plant communities, might explain why most producers are not supplying white salt.

**Table 4.** Macromineral concentrations found in common forages collected on winter range (*n* = 12 ranches)^*a*^

Item	Blue grama	Needle-and-thread	Prairie junegrass	Prairie sandreed	Sand dropseed	Western wheatgrass	Indian ricegrass
Ca, %	0.29 ± 0.02	0.43 ± 0.08	0.29 ± 0.03	0.48 ± 0.10	0.33 ± 0.00	0.53 ± 0.08	0.63 ± 0.21
P, %	0.04 ± 0.01	0.03 ± 0.01	0.05 ± 0.00	0.14 ± 0.03	0.06 ± 0.01	0.06 ± 0.03	0.05 ± 0.02
K, %	0.12 ± 0.04	0.23 ± 0.20	0.12 ± 0.04	0.44 ± 0.03	0.15 ± 0.01	0.18 ± 0.08	0.18 ± 0.08
Mg, %	0.06 ± 0.01	0.08 ± 0.02	0.06 ± 0.01	0.10 ± 0.03	0.08 ± 0.00	0.10 ± 0.02	0.12 ± 0.01
S, %	0.07 ± 0.02	0.08 ± 0.01	0.05 ± 0.01	0.11 ± 0.01	0.08 ± 0.01	0.09 ± 0.02	0.10 ± 0.01
Na, %	0.05 ± 0.01	0.07 ± 0.03	0.05 ± 0.00	0.04 ± 0.01	0.05 ± 0.01	0.07 ± 0.03	0.07 ± 0.03
	Wyoming big sagebrush	Rubber rabbitbrush	Gardner’s saltbush	Shadscale saltbush	Silver sagebrush	Winterfat	
Ca, %	0.67 ± 0.13	1.29 ± 0.37	1.98 ± 0.46	2.27 ± 0.51	1.00 ± 0.12	2.27 ± 0.50	
P, %	0.21 ± 0.05	0.07 ± 0.02	0.13 ± 0.02	0.08 ± 0.03	0.17 ± 0.01	0.10 ± 0.01	
K, %	1.07 ± 0.13	0.52 ± 0.37	1.28 ± 0.40	2.67 ± 0.56	0.91 ± 0.08	0.78 ± 0.11	
Mg, %	0.17 ± 0.02	0.27 ± 0.10	0.91 ± 0.36	1.13 ± 1.10	0.20 ± 0.02	0.56 ± 0.11	
S, %	0.19 ± 0.05	0.14 ± 0.04	0.55 ± 0.21	0.46 ± 0.22	0.27 ± 0.01	0.16 ± 0.04	
Na, %	0.09 ± 0.05	0.11 ± 0.11	6.03 ± 0.65	4.30 ± 1.52	0.07 ± 0.01	0.09 ± 0.01	

^*a*^Means ± SD on a dry matter basis.

Micromineral concentrations of grass and shrub species are summarized in Table 5. Depending on the dietary concentration of S and Mo, the absorption and physiological bioavailability of Cu will be altered (Underwood, 1981). Copper toxicity can be reached at 15 mg/kg when diets contain normal S and Mo levels (0.18% and 0.5 mg/kg, respectively; NRC, 2007). The highest concentration of Cu was in big sagebrush at 10.37 mg/kg (Table 5). Zinc functions in reproduction (Masters and Fels, 1980), growth (Underwood, 1981), immune function (NRC, 2007), and wool growth (White et al., 1994). Forty-seven percent of producers were utilizing a complete trace mineral (Table 1); however, silver sagebrush had the highest mean concentration of Zn at 53.45 mg/kg (Table 5), which is greater than the NRC (2007) requirement of 20 to 39 mg/kg, yet below the maximum tolerable level at 300 mg/kg. Further suggesting in a dominant shrub winter range, shrubs may provide adequate Zn but are highly dependent on the forage species consumed. Selenium is needed for growth and fertility and when deficient results in white muscle disease (Underwood, 1981). Gardner’s saltbush had the highest level of Se at 3.77 mg/kg. Selenium is regulated by the Food and Drug Administration resulting in complete feeds and supplements not exceeding 0.3 ppm and 0.7 mg/kg per head per day (NRC, 2007), respectively.

**Table 5.** Micromineral concentrations found in common forages collected on winter range (*n* = 12 ranches)^*a*^

Item	Blue grama	Needle-and-thread	Prairie junegrass	Prairie sandreed	Sand dropseed	Western wheatgrass	Indian ricegrass
Zn, mg/kg	18.03 ± 8.2	13.80 ± 5.8	20.13 ± 4.8	43.10 ± 0.4	17.25 ± 2.3	23.39 ± 9.9	26.40 ± 7.5
Se, mg/kg	0.29 ± 0.8	0.11 ± 0.1	0.28 ± 0.1	1.00 ± 0.8	0.30 ± 0.1	0.36 ± 0.3	0.36 ± 0.3
Mn, mg/kg	25.00 ± 7.6	21.67 ± 2.1	36.33 ± 9.7	60.50 ± 30.4	28.00 ± 1.4	35.13 ± 8.8	45.14 ± 23.7
Cu, mg/kg	0.50 ± 0.0	2.97 ± 2.1	2.40 ± 1.2	4.15 ± 0.8	3.75 ± 0.6	3.10 ± 1.1	3.54 ± 1.1
Mo, mg/kg	1.61 ± 1.5	1.58 ± 0.7	2.39 ± 2.6	0.61 ± 0.1	0.90 ± 0.2	3.02 ± 2.8	2.25 ± 1.1
Fe, mg/kg	264.0 ± 83	488.3 ± 122	329.0 ± 137	296.0 ± 116	228.5 ± 7.8	685.7 ± 252	1,170 ± 438
Co, mg/kg	0.20 ± 0.0	0.22 ± 0.04	0.24 ± 0.07	0.20 ± 0.00	0.20 ± 0.00	0.27 ± 0.10	0.55 ± 0.23
	Wyoming big sagebrush	Rubber rabbitbrush	Gardner’s saltbush	Shadscale saltbush	Silver sagebrush	Winterfat	
Zn, mg/kg	46.20 ± 34.7	28.15 ± 12.0	34.19 ± 12.5	34.00 ± 19.9	53.45 ± 11.5	40.20 ± 14.7	
Se, mg/kg	0.90 ± 0.8	0.61 ± 0.7	3.77 ± 4.9	1.27 ± 2.3	1.22 ± 0.9	0.35 ± 0.5	
Mn, mg/kg	41.18 ± 18.4	53.50 ± 17.9	91.50 ± 29.6	119.8 ± 55.2	25.50 ± 8.49	226.5 ± 129	
Cu, mg/kg	10.37 ± 2.3	6.90 ± 1.8	5.21 ± 0.6	4.77 ± 2.6	7.55 ± 0.4	6.40 ± 3.5	
Mo, mg/kg	1.25 ± 0.5	1.51 ± 1.0	1.36 ± 0.5	1.77 ± 1.6	0.47 ± 0.4	1.08 ± 0.6	
Fe, mg/kg	229.0 ± 82.4	421.5 ± 198.5	1,126 ± 228.3	353.5 ± 112.7	107.0 ± 8.49	4,643 ± 3,775	
Co, mg/kg	0.35 ± 0.44	0.20 ± 0.10	0.40 ± 0.16	0.20 ± 0.00	0.20 ± 0.00	1.93 ± 1.91	

^*a*^Means ± SD on a dry matter basis.

## IMPLICATIONS

Results from this study provide insights on mineral element concentrations in shrub and grass species collected on Wyoming winter range. Significant differences of CP, Ca, P, K, Mg, S, Na, Zn, Se, Mn, Cu, and Mo concentrations between shrub and grass species were documented with a clear nutritional advantage for sheep having access to shrubs in the winter. This information provides insight for Wyoming sheep producers to aid in the construction of efficient supplement management decisions relative to the rangeland plant community they have available for sheep, and in light of the producers (42%) not providing a complete trace mineral mix to their ewes throughout the year. Forage mineral concentrations are extremely variable across Wyoming winter ranges and may result in clinical and subclinical deficiencies under extensive production settings leading to economic loss to the producer. However, deficiencies may be more common when sheep are grazing winter range without a significant shrub component in the plant community.



*Conflict of interest statement*. None declared.
